# Impact of Type of Lignin, Alkaline and Lignosulfonate, on Release and Soil Adsorption of Methoxyfenozide Delivered with Lignin-*graft*-PLGA Nanoparticle

**DOI:** 10.1021/acsomega.6c03156

**Published:** 2026-09-11

**Authors:** Sumit Libi, Carlos E. Astete, Eban Hanna, Fannyuy Kewir, Cristina M. Sabliov

**Affiliations:** † Biological & Agricultural Engineering, Louisiana State University, Baton Rouge, Louisiana 70803, United States; ‡ Department of Chemical and Biomolecular Engineering, Johns Hopkins University, Baltimore, Maryland 21218, United States; § Institute for NanoBiotechnology, Johns Hopkins University, Baltimore, Maryland 21218, United States

## Abstract

The intensive application of pesticides in agriculture suffers from poor dispersion, runoff, and rapid degradation, which has raised serious environmental and economic concerns. This study investigates the use of lignin-based nanoparticles (LNPs) as sustainable carriers for the controlled delivery of methoxyfenozide (MFZ), enhancing delivery efficiency and reducing environmental impact. Core–shell nanoparticles were synthesized using alkaline lignin (ALN) and lignosulfonate (SLN), both grafted with poly­(lactic-*co*-glycolic acid) (PLGA) biopolymers. Both systems achieved 3 wt % MFZ loading with entrapment efficiencies exceeding 50% (52.7% for SLNPs and 56.3% for ALNPs), yielding spherical, negatively charged particles of different sizes: SLNPs (250.3 ± 9.2 nm) and ALNPs (165 ± 0.4 nm). Release studies evaluated under two pH and temperature conditions demonstrated that the lignin type used influenced NP size and active component release, correlating with differences in the functional group composition (sulfonic, carboxylic, and phenolic groups) of ALN and SLN. Soil adsorption experiments showed enhanced MFZ adhesion to soil when delivered via LNPs (80% MFZ adsorbed compared to 40% when delivered in free form), potentially reducing runoff and overapplication. Overall, these findings identify key governing parameters, providing a mechanistic basis for engineering lignin-based nanoparticles with customizable release profiles and soil adsorption as pesticide delivery systems.

## Graphical abstract

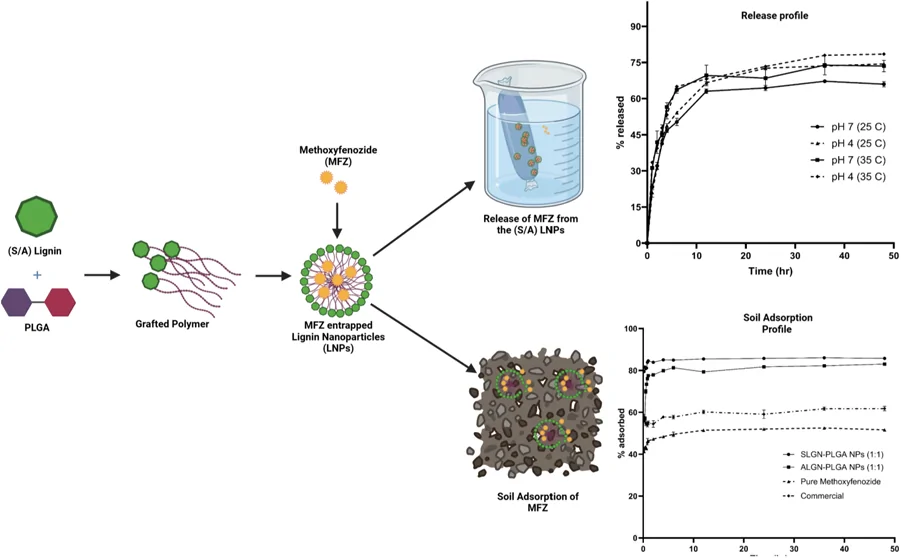

## Introduction

It is estimated that pests and insects have been responsible for 18–20% of annual crop losses worldwide, amounting to over USD 470 billion.^1^ Pesticides are used to protect crops from pests and improve the quality and quantity of agricultural production.^2,3^ However, due to degradation, poor dispersion of traditional formulations, water runoff, and other factors, 90% of applied pesticides never reach their intended target.^4,5^ Hence, heavy application of a wide range of agrochemicals has become a common strategy to control the damage caused by fungi, pests, and weeds.^5,6^ Their overapplication increases the cost of crop production and adversely impacts the environment and human health.^7,8^ The development of environmentally friendly agrochemical formulations that can boost production without adverse effects on the environment or animal and human health is a central goal of sustainable agriculture.^3,9^

Nanotechnology can play a crucial role in addressing these challenges.^10–20^ Agrochemical-loaded nanoparticles (NPs) can be engineered to target specific pests or diseases,^10–15,21,22^ to efficiently release the active ingredient over time for prolonged availability,^2,3,19,23–29^ and to prevent degradation of pesticides,^2,26^ hence increasing the agrochemical’s effectiveness. Consequently, the pesticide usage amount and its environmental impact are reduced. NPs synthesized from biopolymers specifically possess several advantages over other nanodelivery systems, such as biodegradability, biocompatibility, and ease of production.^30^

To date, various natural biopolymers, such as chitosan, sodium alginate, cellulose derivatives, polypeptides, and lignin, have been used to formulate NPs as delivery systems.^2,8,23,25,31–38^ Among these biopolymers, lignin, an underutilized byproduct of the pulping process and the second most abundant biopolymer after cellulose, is one of the most promising and attractive options for agricultural applications.^6,39–41^ The various physicochemical properties and features, such as amphiphilicity, high thermal stability, antioxidant activity, and the presence of different reactive groups, such as phenolic hydroxyl, carbonyl, and sulfonyl, have triggered a high interest in the use of lignin to develop various biobased products.^2,5,8,35,40–45^ Further, natural degradation of lignin into humus enhances soil fertility and reduces urea nitrification, which is beneficial for crop growth and development,^6,39,46^ making it an even more valuable resource.

Various lignin-based nanocarriers have been developed to enhance the delivery and effectiveness of agrochemicals. Pyraclosrobin encapsulated in kraft methacrylated lignin nanocarriers was synthesized with 90% encapsulation efficiency and used to reduce Esca disease symptoms in grapevines.^43^ Different fungicides have been encapsulated in spermine/spermidine cross-linked nanocarriers with high efficiency.^35^ Lignin-chitosan nanocarriers were used to deliver natural fungicides against *N. parvum*.^46^ Lignosulfonate-modified epoxy resin nanocarriers demonstrated slower release, improved soil mobility, and absorption in plant roots of abamectin.^47^ Avermectin-loaded lignin-based azo polymer demonstrated controlled release, xylanase-responsive lignin-xylan nanospheres demonstrated higher release rates in the presence of xylanase, lignosulfonate-bromide nanospheres exhibited different release rates and enhanced antiphotolysis activity. pH-responsive release has been shown for cyhalothrin-loaded core–shell nanostructures and lignin-based chlorpyrifos nanogel carriers, and diuron-loaded subabul stem lignin nanoformulations.^35,43,47,48^ All of the prior studies have examined individual lignin sources (kraft lignin, lignosulfonate, alkaline lignin) or tested different agrochemicals across different lignin types in separate investigations. Also, most prior studies optimize for a single outcome: either controlled release kinetics or soil retention, but do not address release and adsorption simultaneously. No study, to the authors’ knowledge, has been published on the comparison of how different lignin types—with distinct functional group compositions grafted on PLGA—affect both release kinetics and soil retention of nanodelivered agrochemicals.

In this research, the main objective was to determine the effect of the type of lignin, alkaline and lignosulfonate, on methoxyfenozide (MFZ) release and soil adsorption when delivered with lignin-based NPs (LNPs). Methoxyfenozide (MFZ) is commonly used to control the growth and development of lepidopteran pests; it is considered to be relatively safe for nontarget organisms and to have low aquatic toxicity and low soil persistence; hence, it was selected for this study.^49,50^ In this study, MFZ-loaded LNPs were synthesized from two different types of lignin: sodium lignosulfonate (SLN) or alkaline lignin (ALN). Lignosulfonates and alkaline lignin have distinct properties. The presence of various hydroxyl, carboxylic, and sulfonic groups in different amounts in ALN and SLN^51^ provides the opportunity to engineer LNPs of tunable properties and functionalities, in terms of size, release kinetics, and soil adsorption of nanodelivered MFZ. Lignosulfonates typically have higher S content due to the sulfonation process and are more water-soluble than alkaline lignin. Sulfonic acid groups (−SO_3_H, p*K*_a_ ∼ 1–2), together with carboxylic acid groups (−COOH, p*K*_a_ ∼ 4–6),^52^ phenolic hydroxyl groups (−OH, p*K*_a_ ∼ 9–10), and aliphatic hydroxyl groups (−OH, p*K*_a_ ∼ 12.5–17)^53^ greatly improve the aqueous solubility of lignosulfonate.^52^ Both SLNs and ALNs have been used to engineer controlled-release formulations for pesticides.^42^

The novelty of the lignin nanodelivery system proposed herein is that it is formed from an amphiphilic biopolymer synthesized by grafting SLN or ALN onto a synthetic, biodegradable polymer poly­(lactic-*co*-glycolic acid) (PLGA) at 1:1 w/w (S/A)­LN/PLGA to develop a core–shell nanoparticle. The impact of the type of lignin on the formation of core–shell nanoparticles, the controlled release of the hydrophobic MFZ entrapped in the core of the particles, and MFZ soil adsorption were assessed. The release profile was measured using the dialysis method^54^ and analyzed by high-performance liquid chromatography (HPLC) at two pH values (4 and 7) and two different temperatures (25 and 35 °C). The sorption experiments were performed by following the OECD guidelines for adsorption–desorption using the batch equilibrium method^55^ to evaluate the effect of the type of LNPs on the adsorption of pesticides by soil, as a measure of the ability of the nanodelivery systems to curb the environmental impact of the pesticide.

## Materials and Methods

### Chemicals

Sodium lignosulfonate (SLN) and alkaline lignin (ALN) were purchased from TCI (Portland, OR); oxalyl chloride, dimethylformamide (DMF), ethyl ether, dimethyl sulfoxide (DMSO), and HPLC-grade acetonitrile were purchased from Thermo Fischer Scientific (Waltham, MA); dichloromethane (DCM) and poly lactic-*co*-glycolic acid (1:1) (35,000–45,000 Da) (PLGA) were purchased from Sigma-Aldrich (St. Louis, MO); and Methoxyfenozide (MFZ) was obtained from AccuStandard (New Haven, CT). All chemicals used were laboratory grade. All aqueous nanoparticle synthesis solutions and HPLC analyses were made with ultrapure deionized (DI) water (Millipore, Milli-Q, Barnstead International, Dubuque, IA). The MFZ standard stock solution was prepared in acetonitrile, and the serial dilutions were prepared by dilution with acetonitrile.

## Polymer Synthesis and Nanoparticle Formation

### Polymer Synthesis

(S/A) LN-*g*-PLGA polymers were prepared as described by Astete et al.^54,56^ with minor modifications. Briefly, 1 g of PLGA was dissolved in 100 mL of DCM at room temperature in a 3-neck round-bottom flask. Following complete dissolution, 110 μL of oxalyl chloride was added dropwise, followed by 4 mL of DMF as a catalyst. The reaction mixture was stirred at room temperature for 4 h. The excess DCM was evaporated using a rotavapor (Buchi Corporation, New Castle, DE) at 300 mbar at 30 °C. The resulting polymer was dissolved in 25 mL of DMSO and added dropwise to 1 g of LN dissolved in 20 mL of DMSO to get 1:1 mass LN:PLGA ratio. After the overnight reaction, the mixture was precipitated in 200 mL of ethyl ether to precipitate the LN-*g*-PLGA polymer. The precipitated polymer was washed multiple times with ethyl ether and dissolved in 20 mL of DCM. The suspended polymer was washed with DI water to remove any unreacted LN. The excess DCM was evaporated, and the resulting polymer was frozen at −80 °C for 24 h before lyophilization using a freeze-dryer (Labconco FreeZone 2.5 plus, Labconco, Kansas City, MO).

### Nanoparticle Formation

Empty (S/A)­LNPs were prepared using the emulsion evaporation method as described by Astete et al.^56^ An organic phase was prepared by dissolving 500 mg of (S/A)­LN-*g*-PLGA polymer in 7 mL of ethyl acetate at room temperature. This organic phase was added to 70 mL of DI water (aqueous phase) under continuous mixing for ∼10 min. The resulting mixture was homogenized with a microfluidizer (Microfluidics Corp., Westwood, MA) at 30 kPsi and 4 °C. Following emulsification, ethyl acetate was evaporated under vacuum at 30 °C for 1 h. Trehalose (1:1 w/w) was added as a cryoprotectant, and the samples were frozen before lyophilization at −80 °C for 2 days.

For the MFZ-loaded LNPs, 40 mg of MFZ and 500 mg of (S/A)­LN-*g*-PLGA biopolymer were dissolved in 7 mL of ethyl acetate at room temperature under stirring to form the organic phase. The organic suspension was added dropwise to 70 mL of aqueous phase (DI water) and homogenized as described above. Ethyl acetate was evaporated under vacuum at 30 °C for 1 h, and samples were processed as described above.

## Characterization of the NPs

### Size, Size Distribution, and Zeta Potential

The average particle size, polydispersity index, and zeta potential of the synthesized NPs were determined by dynamic light scattering (DLS) using a Malvern Zetasizer ZS (Malvern Analytical, Westborough, MA). Freshly made (S/A)­LNP suspensions (1 mg mL^–1^ in DI water) were analyzed at 25 °C. The PDI and zeta potential indicated the size distribution and stability of the NPs.

### Morphology

The morphology of the (S/A)­LNPs was examined with a transmission electron microscope (TEM). Samples were prepared by placing a droplet of resuspended NP suspension (1 mg mL^–1^) on a carbon-coated copper grid, staining with a contrast agent (0.5% uranyl acetate), and air-drying for 10 min. Samples were imaged with a TEM JEM-1400 (Jeol USA Inc., Peabody, MA).

### MFZ Loading and Entrapment Efficiency

To evaluate the loading content and entrapment efficiency of MFZ, the (S/A)­LNPs suspensions (5 mg mL^–1^ in DI water) were filtered through a 0.22 μm PTFE membrane. The filtrate was diluted with 400 μL of acetonitrile to extract MFZ entrapped in LNPs, and MFZ content was analyzed using HPLC with a C18 analytical column (150 mm × 4.6 mm ID × 5 μm), and a diode-array detector. An injection volume of 20 μL was used, and the mobile phase consisted of acetonitrile–water (90/10, v/v), and the flow rate was set at 0.75 mL/min. The column temperature was set to 25 °C. Based on the standard curve data generated from seven standard MFZ dilutions, the retention time was approximately 8 min.

The entrapment efficiency and loading capacity were calculated using the following equations

1 $$entrapment\;efficiency\;\left(\%\right)=\frac{m}{m_{0}}\times 100\%$$

2 $$loading\;capacity\;\left(LC\;\%\right)=\frac{m}{m_{np}}\times 100\%$$

where *m* is the mass of MFZ loaded in (S/A)­LNPs, *m*_0_ is the mass of MFZ added to the synthesis, and *m*_np_ is the mass of NPs synthesized.

### Realese Profile

MFZ release from (S/A)­LNPs was evaluated using the dialysis method, as described by Mendez et al.^54^ 10 mL of the MFZ-loaded (S/A)­LNPs suspension (10 mg mL^–1^) was loaded into the dialysis membrane (*M*_WCO_ = 12–14 kDa, Fisherbrand, USA) and immersed in 800 mL of phosphate-buffered saline (PBS). Release experiments were conducted at two different pH values of 4 and 7, at room temperatures of 25 or 35 °C. Aliquots (100 μL) were withdrawn from the dialysis bag at specified time points (1, 2, 3, 4, 6, 12, 24, 36, and 48 h) until 100% release or a plateau was achieved; and each time an aliquot was taken, it was replaced with the same volume of DI water. The PBS solution was replaced every 24 h to maintain sink conditions. The sample aliquots were analyzed by HPLC as described above to determine MFZ loading and entrapment. The release study was performed in duplicate for ALNPs and in triplicate for SLNPs.

### Release Kinetics

The release data of (S/A)­LNPs were fitted to three different kinetic models: first-order, Korsmeyer–Peppas, and Hixson–Crowell. Each model was evaluated under four conditions (pH 7 and pH 4, each at 25 and 35 °C), and the *R* ^2^ value was used to assess the goodness of fit.

The first-order model, which describes a release rate dependent on the remaining concentration of the analyte, is expressed as

3 $$\log C_{t}=\log C_{0}-\frac{\mathrm{Kt}}{2.303}$$

where *C_t_* is the concentration remaining at time *t*, *C*_0_ is the initial concentration, and *K* is the first-order rate constant.

In the Korsmeyer–Peppas model, the release of the agrochemical from the polymeric matrix system is governed by diffusion, swelling/NPs erosion, or a combination of both mechanisms, and it is given by

4 $$\frac{M_{t}}{M_{\infty }}=Kt^{n}$$

where *M_t_*/*M*_∞_ is the fraction of solute released at time *t*, *K* is the release rate constant, and *n* is the release exponent, whose magnitude defines the transport mechanism (Fickian diffusion if *n* ≤ 0.43, swelling/NPs erosion if *n* ≥ 0.85, or a combination of both mechanisms if 0.43 < *n* < 0.85).

The Hixon–Crowell model describes the release from systems undergoing dissolution or surface erosion, with release kinetics determined by changes in particle surface area and diameter, and is expressed as

5 $${Q_{o}}^{1/3}-{Q_{t}}^{1/3}=\mathrm{Kt}$$

where *Q*_0_ is the solid’s initial weight, *Q_t_* is the amount of solid remaining at time *t*, and *K* is the dissolution rate constant. The rate constants, release exponents, and corresponding *R* ^2^ values obtained for each model were calculated for all formulations under all tested conditions.

### Soil Characterization

The soil samples were obtained from the LSU AgCenter plant material center greenhouse located at Ben Hur Drive, Baton Rouge, LA. Sieve analysis and hydrometer tests were performed at Eustis Engineering to determine the soil composition. The soil characterization analysis showed that the field soil sample was composed of 21.2% sand, 58.3% silt, and 20.5% clay (Table 1). A soil mixture containing 90% sand, 7% silt, and 3% clay was prepared by combining field soil with masonry sand at a 1:7 ratio.

**Table 1:** Properties of the Soil Used in This Study

soil	% sand	% clay	% slit
field soil	21.2	20.5	58.3
field soil: sand (1:7)	90.15	2.56	7.29

### Sorption Profile

MFZ sorption on soil was determined using the standard batch equilibrium method following the OECD guidelines.^55^ Soil suspensions in 0.01 M CaCl_2_ were prepared in 15 mL PTFE centrifuge tubes, with a soil:solution ratio of 1:2 (w/w) and pre-equilibrated on a shaker for 14 h. The soil suspensions were spiked with either pure MFZ suspension, commercial pesticides, or an MFZ-loaded (S/A)­LNPs suspension, to achieve approximately 1.5 mg kg^–1^ MFZ in the soil. After spiking, the soil suspensions were shaken continuously. The MFZ sorption was subsequently measured at 10 min intervals for the first hour and at 2, 3, 6, 12, 24, 36, and 48 h. At each point, samples were centrifuged at 3000*g* for 30 min (Fisher Scientific, Pittsburgh, PA), and a 100 μL aliquot of the supernatant was analyzed to determine the MFZ concentration remaining in solution after adsorption, using the same method described above.

### Adsorption Kinetic Modeling

The adsorption kinetics of MFZ onto soil were fitted to pseudo-first-order (PFO) and pseudo-second-order (PSO) kinetic models, and the adsorption rate constants were determined for both models. The best-fit model was assessed by using the *R* ^2^ value.

The PFO model assumes that the rate of adsorption is proportional to the number of unoccupied adsorption sites^57^ and is expressed as

6 $$\ln\left(q_{e}-q_{t}\right)={\ln q}_{e}-k_{1}t$$

where *q*_e_ (mg g^–1^) is the adsorption capacity at equilibrium, *q_t_* (mg g^–1^) is the adsorption capacity at time *t* (h), and *k*_1_ is the pseudo-first-order rate constant.

The PSO model assumes that the rate-limiting step is due to chemisorption^57^ and is expressed as

7 $$\frac{t}{q_{t}}=\frac{1}{k_{2}q_{e}^{2}}+\frac{t}{q_{e}}$$

where *k*_2_ (g mg^–1^ h^–1^) is the pseudo-second-order rate constant and remaining parameters as defined above.

The kinetic constants were determined from the slopes and intercepts of the respective linear plots, and the *R* ^2^ values of the two models were compared to identify the best model that described the adsorption behavior of MFZ on the soil.

### Statistical Analysis

Statistical comparisons between the (S/A)­LNPs were performed using an unpaired two-tailed Student’s t-test (GraphPad Prism, GraphPad Software, San Diego, CA, USA). Nanoparticle size was compared using independent *t* tests, and release profiles were compared using paired *t* tests under different conditions (pH and temperature) for the same nanoparticle system. Data are presented as mean ± standard deviation (SD). Statistical significance was set at *p* < 0.05.

## Results and Discussion

### (S/A)­LN-*g*-PLGA Polymer

FT-IR analysis was performed to confirm the conjugation of (S/A)­LN to PLGA. The FT-IR spectra exhibited all of the characteristic absorption bands corresponding to hydroxyl groups associated with phenolic and carboxylic acids in the region (1900 to 800 cm^–1^). Distinct characteristic peaks for lignin at 1600 and 1505 cm^–1^, as well as the characteristic carbonyl stretching vibration for PLGA at 1750 cm^–1^ in the biopolymer (Figure 1), were observed, consistent with those previously reported by Astete et al.^56^ In addition, the presence of an absorption band at 620–660 cm^–1^, attributed to the sulfonic groups, confirmed the higher abundance of sulfonic groups (0.235 in the integral area) in the SLGN-*g*-PLGA polymer compared to the ALGN-*g*-PLGA polymer (0.053), affecting the polymer solubility, nanoparticle size, and MFZ release from the synthesized nanoparticles.

**Figure 1:**
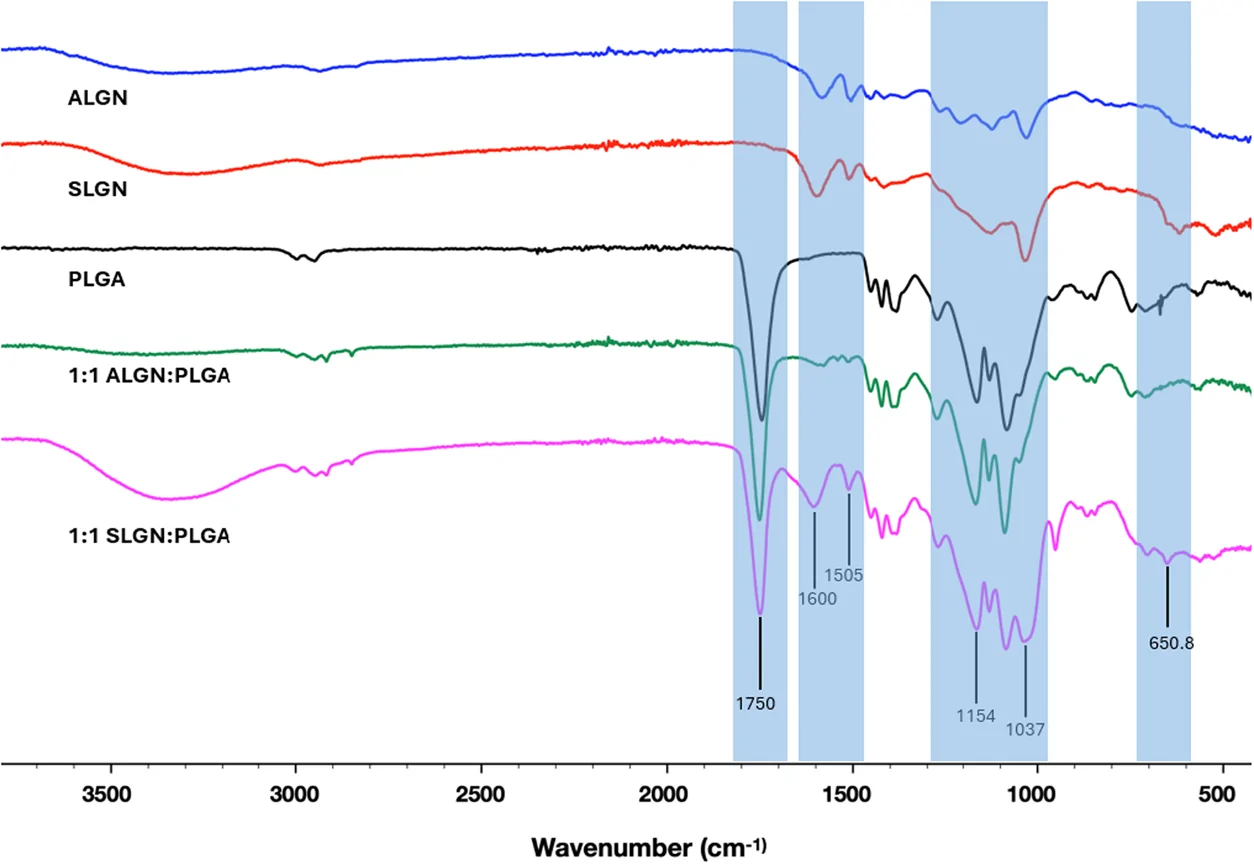
FT-IR spectrum for ALGN, SLGN, PLGA, and (S/A)­LGN-*graft*-PLGA polymers: ALGN-*g*-PLGA (1:1), SLGN-*g*-PLGA (1:1) from top to bottom.

### Nanoparticle Characterization

The grafted polymers were further used to form empty (S/A)­LNPs and (S/A)­LNPs with entrapped MFZ. TEM images confirmed that both SLNPs and ALNPs were spherical (Figure 2). DLS analysis showed that the nanoparticles were monodispersed as indicated by the polydispersity index (PDI < 0.1) and had a highly negative zeta potential (ζ) when dispersed in deionized water (Table 2). The average hydrodynamic diameter of the MFZ-loaded SLNPs was 250.3 ± 9.2 nm, whereas the MFZ-loaded ALNPs were significantly smaller, with an average diameter of 165.5 ± 0.4 nm (independent *t*-test, *t* = 28.87, *p* < 0.0001) (Table 2).

**Figure 2:**
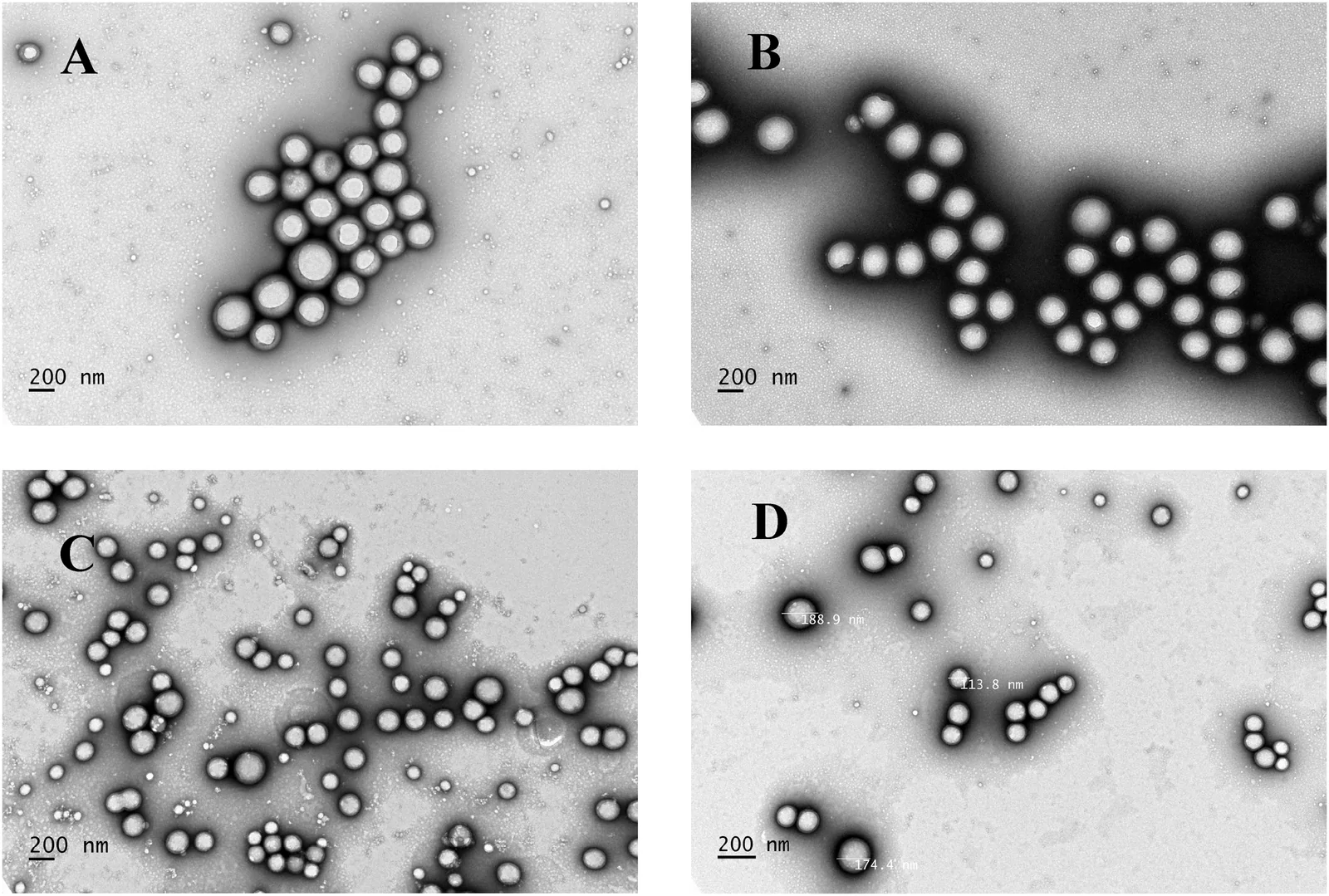
TEM images of (A) empty SLNPs; (B) MFZ-loaded SLNPs; (C) empty ALNPs; and (D) MFZ-loaded ALNPs.

**Table 2:** Size Distribution, PDI, Zeta Potential of Empty and Loaded (S/A)­LNPs as Measured by Dynamic Light Scattering and Entrapment Efficiency (EE) and Loading Capacity (LC) of Loaded NPs

nanoparticles (polymer ratio w-w)	size (nm)	PDI	Zeta (ζ) (mV)	EE (%)	LC (%)
empty SLNPs (1:1)	249.5 ± 3.2	0.05 ± 0.01	–64.5 ± 1.2	N/A	N/A
MFZ-loaded SLNPs (1:1)	250.3 ± 9.2	0.08 ± 0.05	–53.0 ± 1.3	52.7	2.6
empty ALNPs (1:1)	198.8 ± 4.6	0.12 ± 0.09	–77.0 ± 2.6	N/A	N/A
MFZ-loaded ALNPs (1:1)	165.5 ± 0.4	0.07 ± 0.05	–67.4 ± 3.3	56.3	3.2

The difference in size can be attributed to the functional groups present in SLNPs. The electrostatic repulsion between the sulfonate groups prevented the formation of a tight compact structure of the SLNPs.^51^ ALN, more hydrophobic and less charged, corresponded to smaller-sized ALNPs.^58^ In addition, all of the NPs exhibited high negative ζ potential values, contributing to the colloidal suspension’s stability. The ζ potentials of the empty freshly made NPs were −64.5 ± 1.2 mV and −77.0 ± 2.6 mV for SLNPs and ALNPs, respectively, and the ζ potentials of the MFZ-loaded NPs were −53.0 ± 1.3 mV for SLNPs and −67.4 ± 3.3 mV for ALNPs under neutral conditions (Table 2). The complete physicochemical characteristics of the NPs are summarized in Table 2.

In contrast to ALNPs, as indicated earlier, SLNPs have a higher number of sulfonic groups in addition to hydroxyl and carboxylate groups, contributing to the increased charge density, enhanced hydrophilicity, and formation of an extended hydration shell.^59,60^ Image analysis of the SLNPs and ALNPs showed that the outer shell of the SLNPs was double the size of the ALNPs’ outer shell, formed by the lignin polymer. A decrease in the ζ potential values of both (S/A)­LNPs was observed when the pH was reduced from 7 to an acidic pH of 4, as expected due to the partial protonation of the carboxylic groups.

The entrapment efficiency exceeded 50% for all NPs, with 52.7% for SLNPs and 56.3% for ALNPs. These values are within the published entrapment efficiencies for lignin-based nanocarrier systems encapsulating agrochemicals. Literature reports show that EE values for such systems vary broadly from 30% to 95%, depending upon the lignin type used, NPs formation process, morphology, and entrapped active ingredient properties.^43,61^ The reported EEs fall well within the range compared with other lignin-based alkyl-modified lignosulfonates, which achieved 50% EE with avermectin,^62^ and hollow spheres from lignin-based azo polymer achieved 57.9–67% EE of avermectin,^43^ dioxane lignin achieved 74% EE of diuron,^63^ and several pesticide-lignin systems report 61–74% EE values.^61,64,65^

The loading capacity ranged from 2.6 to 3.2% for all NPs synthesized. In other words, 25–35 μg MFZ was loaded per 1 mg of (S/A)­LNPs. The loading capacity of the SLNPs was similar to the LNPs previously reported by Mendez et al.^54^

### Methoxyfenozide Release

The release of MFZ from (S/A)­LNPs was measured at pH 4 and 7 at two different temperatures, 25 and 35 °C. SLNPs and ALNPs showed a prolonged MFZ release, with different release profiles. The cumulative release was faster at higher temperatures, similar to what has been reported in the literature,^54^ and reached a plateau after 24 h for both systems. It can be observed from Figure 3 that the MFZ release profile was different for the two types of LNPs, and pH affected MFZ release from SLNPs but not from ALNPs. For SLNPs (n = 3), a relatively short, rapid-release phase was observed in the first 6 h, when 40% of the entrapped MFZ was released, followed by a gradual-release phase from 12 to 48 h at both pH values and temperatures. The cumulative MFZ release was significantly higher at acidic pH (pH 4) compared to the neutral pH (pH 7), with 75% and 68% of MFZ released, respectively, at 48 h (*p* = 0.0271). At pH 7 and 25 °C, cumulative MFZ release reached 50.3% by 6 h, whereas at 35 °C, cumulative release reached 63.6% at the same time point, representing a 26.4% increase at the higher temperature. Similarly, a 20% increase in cumulative release was observed at pH 4 at the higher temperature. By 12 h, the temperature effect was moderately reduced (pH 7:10.4% increase, pH 4:2.4% increase), and by 24 h, cumulative release values differed by only 6.3% at pH 7 and 1% at pH 4, which indicates that elevated temperature accelerates the release in the early stage.

**Figure 3:**
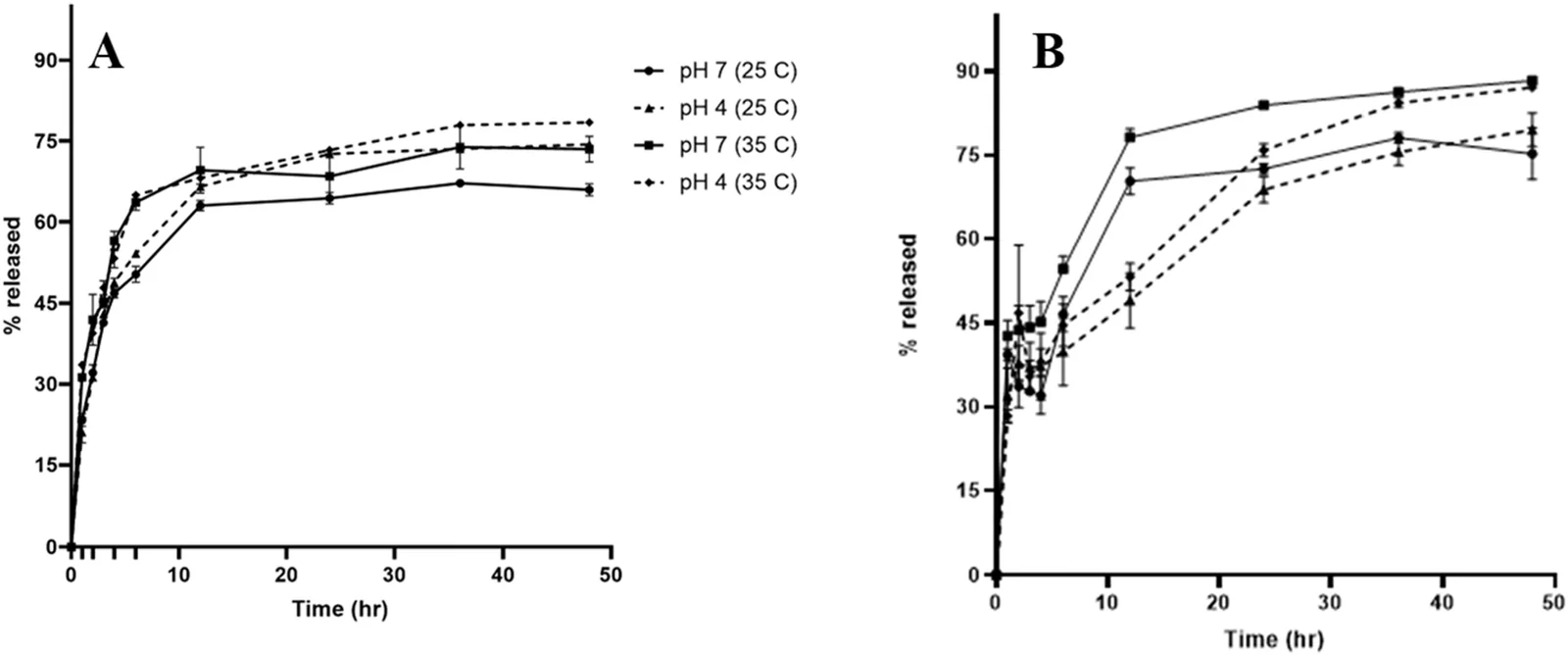
Release profiles for MFZ loaded in (A) SLNPs (*n* = 3) and (B) ALNPs (*n* = 2). Error bars represent ± SD of three measurements.

At pH 7, carboxylic groups are deprotonated along with the permanently ionized sulfonate groups in the SLNPs, which form extensive hydration shells. These hydration layers enhance water organization at the NP interface and create a more stabilized hydrated matrix. As a result, the diffusion of MFZ is governed primarily by diffusion across the hydration shell created by the concentration gradient of MFZ within the core and aqueous medium. At pH 4, the protonation of coexisting carboxyl groups in the lignin shell reduces the charge and structure of the hydration shell, which promotes the faster release of MFZ.^66^ Zhou et al.^67^ have also reported a faster release of doxorubicin hydrochloride at acidic conditions compared to neutral pH from lignin-based NPs and have related pH-based release to shell thickness of NPs. Similarly, Wibowo et al.^68^ have reported on the release under acidic conditions due to protonation of the functional group in SLNPs and a decrease in the hydration layer.

ALNPs demonstrated a biphasic release profile characterized by an initial burst phase followed by sustained release (Figure 3b). It was observed that the cumulative release reached 30–40% within the first 2 h, followed by a plateau phase as it reached 4 h, when the release rate slowed down significantly, followed by a sudden release from 6 to 12 h, with the cumulative release reaching 65% (pH 7) at 25 °C and 80% at 35 °C. At pH 7, cumulative release reached 48.44% by 6 h at 25 °C, whereas at 35 °C, cumulative release was 48.24%, indicating a minimal temperature effect during the early phase. However, by 48 h, the temperature effect became apparent, with a cumulative release of 79.56% at 25 °C increasing to 84.4% at 35 °C, representing a 6.1% increase in cumulative release at elevated temperatures.

Yearla et al.^59^ reported a similar release profile. The initial burst release is possibly due to the rapid release and fast diffusion of loosely bound MFZ from the periphery of ALNPs held by a hydrogen bond between MFZ and hydroxyl and carboxylic groups, followed by slower sustained diffusion of MFZ embedded in the core of the nanoparticles, prompting the second phase of release as observed and suggested in the literature. A similar biphasic release profile with an initial rapid release followed by sustained, slower release has been reported for diuron from subabul stem lignin nanoformulations^63^ and cilazapril from Kraft LNPs.^69^ Overall, the MFZ release from ALNPs in acidic media was similar to that at neutral pH, with no significant difference observed (*p* = 0.3786).

The results of the release of MFZ from both NPs systems were evaluated using three kinetic release models (first-order, Korsmeyer–Peppas, and Hixon–Crowell). In the first-order release model, the release rate is proportional to the concentration gradient. In the Korsmeyer–Peppas model, the release from the polymeric matrix system is governed by Fickian diffusion (*n* ≤ 0.43), swelling/NPs erosion (*n* ≥ 0.85), or a combination of both mechanisms (0.43 < *n* < 0.85). The Hixon–Crowell model describes the release from systems undergoing dissolution or surface erosion, with release kinetics determined by the change in surface area and diameter of the particles. The correlation coefficient (*R* ^2^) value and constants for all three models applied are presented in Table 3.

**Table 3:** Correlation Coefficients and Best Fit for Release of Methoxyfenozide from (S/A)­LNPs for Three Kinetic Models

		SLNPs				ALNPs			
kinetic models	parameters	pH 7 (25 °C)	pH 7 (35 °C)	pH 4 (25 °C)	pH 4 (35 °C)	pH 7 (25 °C)	pH 7 (35 °C)	pH 4 (25 °C)	pH 4 (35 °C)
First-order	*K*	0.0068	0.0055	0.0081	0.0061	0.0109	0.0073	0.0086	0.0093
	*R* ^2^	0.5219	0.4935	0.5241	0.5994	0.4891	0.7434	0.8492	0.7971
Korsmeyer–Peppas	*K*	28.64	36.98	27.22	36.89	20.56	37.15	29.87	28.77
	*n*	0.26	0.21	0.30	0.22	0.35	0.24	0.25	0.28
	*R* ^2^	0.8715	0.83	0.8708	0.9114	0.6042	0.8703	0.819	0.8277
Hixon–Crowell	*K*	0.021	0.023	0.028	0.028	0.045	0.059	0.048	0.059
	*R* ^2^	0.6055	0.5477	0.6812	0.6621	0.6689	0.7826	0.8597	0.8916

As presented in Table 3, *R* ^2^ values suggested that the release kinetics of MFZ from the SLNPs is best described by the Korsmeyer–Peppas model (*R* ^2^ > 0.85), indicating that the release is controlled by diffusion of MFZ from the NPs system. The application of the Korsmeyer–Peppas mathematical model to the release of MFZ at pH 7 (25 °C) from SLNPs yielded the values 28.64 h^–1^, 0.26, and 0.8715 for (*k*), (*n*), and (*R* ^2^), respectively. The high value for the kinetic rate constant suggests that the release was fast.

Based on the *R* ^2^ value for the release of MFZ from ALNPs (Table 3), the Hixon–Crowell model and the Korsmeyer–Peppas model provided the best fit, indicating that the release is consistent with either of the models. Based on the Korsmeyer–Peppas model, the release rate constant (*k*) and release exponent (*n*) were determined to be 20.56 h^–1^ and 0.33 for the release of MFZ at pH 7, 25 °C, and the dissolution rate constant (*K*) was determined to be 0.045 h^–1^ for the Hixon–Crowell model. The dissolution rate constant implies how fast the system dissolves over time and affects the release, suggesting a slow-sustained release.

The Korsmeyer–Peppas model presented n values below 0.45 for both LNPs, further supporting that release from both systems is best described by Fickian diffusion driven by the concentration gradient of MFZ from nanoparticles and medium, consistent with the study done by Andersson et al.^70^ The differences in the *R* ^2^ values between the two systems suggest that while both follow diffusion-based release, the functional group chemistry of each lignin type modulates the release kinetics differently. The higher *R* ^2^ values for the SLNPs indicate more-predictable, purely diffusion-controlled release, whereas the dual-model fit for ALNPs indicates additional possible contributions from dissolution.

A paired *t-*test was used to compare the release of MFZ from (S/A)­LNPs at the two pH values and two temperatures. The pairwise *t-*test concluded that the release of MFZ from SLNPs was significantly different at both pH and temperatures, with *p* < 0.05, whereas for the ALNPs, the *t-*test of the release of MFZ from ALNPs showed a significant difference in release at two temperatures for both pH and no significant difference at the acidic and neutral pH at 25 °C (*p* > 0.05). As shown by the t-test, SLNPs released significantly faster at acidic pH compared with neutral pH, whereas for ALNPs, the two pH levels tested did not make a difference. Release studies demonstrated that both LNP systems responded to the temperature difference (25 and 35 °C). pH-dependent release was significant only for SLNPs but not for ALNPs, reflecting the impact of the distant functional group protonation/deprotonation behavior.

### Soil Adsorption

The adsorption of MFZ in the soil is a dynamic equilibrium process, and the soil adsorption of MFZ using different formulations is an important factor to consider in designing controlled-release systems to curb the environmental impact of the nanodelivered agrochemical. The adsorption data showed that MFZ loaded in (S/A)­LNPs was adsorbed faster than MFZ in the free form and in the commercial pesticide formulation. Almost 80% of the MFZ loaded in (S/A)­LNPs was adsorbed by the soil, whereas only 50–60% of free MFZ and MFZ in the commercial formulation were adsorbed by the soil. This indicates that the LNPs helped with the soil adhesion of MFZ, decreasing runoff of the pesticide and therefore its negative environmental impact. The presence of active sites on lignin with diverse hydroxyl, carboxyl, aliphatic, and phenolic groups has been reported to be responsible for interactions between LNPs and the soil particles,^71^ leading to enhanced adsorption of nanodelivered MFZ. A difference in adsorption profiles between two LNP systems (*p* < 0.0001) can be attributed to the hydration shell thickness.

The larger hydration shell surrounding SLNPs promotes stronger interactions with soil, facilitating the increased retention of NPs and MFZ in the soil.

The adsorption kinetics model was used to describe the rate equation for the liquid–solid adsorption kinetics. The experimental data were fitted using pseudo-first-order (PFO) and pseudo-second-order (PSO) kinetic models (Figure 4b,4c) to determine the rate constants. These kinetic models describe the adsorption rate over time during the entire process of 48 h. PFO kinetics assumes that the adsorption rate depends on the concentration of unoccupied binding sites, whereas PSO assumes that the rate-limiting step is due to chemisorption.^57^

**Figure 4:**
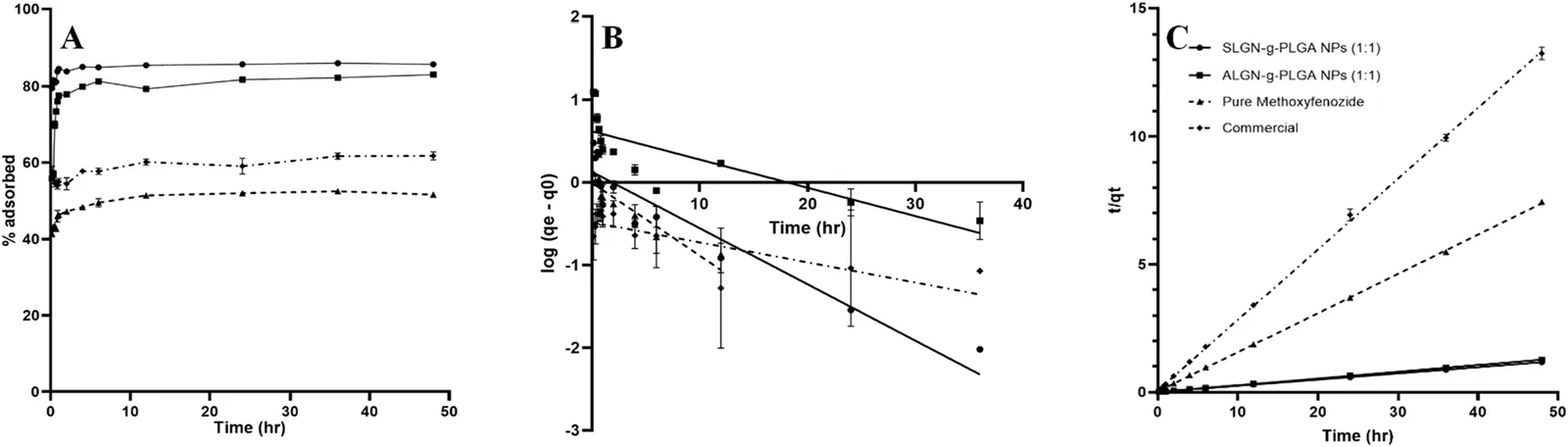
(A) Adsorption kinetics, (B) Pseudo-first-order, and (C) Pseudo-second-order kinetic model for the adsorption of MFZ from MFZ loaded in (S/A)­LNPs, free MFZ, and commercial MFZ pesticide. Error bars represent ± SD of *n* = 3.

The soil adsorption of MFZ exhibited two distinct stages (Figure 4A). In stage 1 (0–1 h), sorption of MFZ to readily available soil binding sites occurred rapidly. This rapid phase achieved approximately 50–70% of the equilibrium adsorption capacity within the first hour. Following the initial rapid sorption, the rate of adsorption of remaining MFZ decreased significantly. In stage 2 (1–12 h), adsorption proceeded slowly as MFZ bound with less accessible soil binding sites within micropores. This achieved the remaining 30–50% adsorption capacity gradually over the 1–12 h time period. The PSO model confirms that chemisorption, a slower chemical binding process, occurred within this time period, indicating that maximum soil uptake capacity was reached. As presented in Table 4, the PFO model showed poor agreement with the experimental data across all of the systems, yielding low *R* ^2^ values for commercial pesticide formulation and free MFZ, and the highest correlation was observed for SLNPs. In contrast, the PSO kinetic model provided an excellent fit to the adsorption data, indicating that this model accurately describes the adsorption behavior.

**Table 4:** Constants and Correlation Coefficients for the Kinetic Models

kinetic models	parameters	SLNPs	ALNPs	Free MFZ	commercial pesticide
Pseudo-first-order	*K*_1_	–0.16	–0.079	–0.202	–0.05
	*q*_e_	1.35	4.17	0.993	0.330
	*R* ^2^	0.8412	0.6267	0.7706	0.3588
Pseudo-second order	*K*_2_	0.76	0.18	1.095	1.39
	*q*_e_	41.15	37.95	6.51	3.61
	*R* ^2^	1	0.9999	0.9998	0.9993

Under PSO, the soil sample showed the highest equilibrium adsorption capacity for SLNPs and ALNPs (*q*_e_ = 41.15 mg/g and *q*_e_ = 37.95 mg/g) compared to the adsorption of the commercial pesticide formulation (*q*_e_ = 6.51 mg/g) and free MFZ (*q*_e_ = 3.61 mg/g). This kinetic parameter demonstrates that the (S/A)­LNPs system exhibits higher affinity and retention in the soil systems compared to free and commercial pesticide formulations.

## Conclusion

In this study, a lignin-based nanoparticle delivery system was proposed to address issues associated with the overuse of pesticides and their negative environmental impacts. This study successfully demonstrated that LNPs synthesized from ALN and SLN grafted with PLGA can serve as promising carriers for a controlled delivery system. The evaluation of two distinct lignin types showed that MFZ can be effectively encapsulated (more than 50% entrapment efficiency, 3 wt % loading capacity) to form spherical core–shell nanoparticles and can govern both release kinetics and soil interactions of nanodelivered MFZ.

Evaluation of the release profiles of MFZ from LNPs made from alkaline and lignosulfonate, carried out under different pH conditions (pH 7 and pH 4) and temperatures (25 and 35 °C), showed that these LNPs were pH- and temperature-responsive, with varying release rates based on the type of lignin used for their synthesis. This indicates that the presence of different functional groups of lignin played a crucial role in the controlled release of the pesticide. These physicochemical differences between SLNPs and ALNPs provide a framework for designing formulations tailored to specific agricultural scenarios. Based on the Korsmeyer–Peppas model, it can be concluded that the MFZ was released mainly through diffusion, with some erosion contribution for ALNPs. The adsorption study revealed that LNPs improved the adhesion of MFZ to the soil. The LNPs system’s increased soil sorption can prevent runoff and overuse. The finding that 80% of LNPs delivered MFZ binds to soil (compared to 50% for free pesticide) has direct economic and environmental implications: reduced loss of pesticide runoff that could permit lower application rates, while maintaining agronomic efficacy.

Despite these promising results, several limitations of this study should be acknowledged. This study was conducted using a single model pesticide (MFZ) to establish a reproducible experimental framework for evaluating the release and adsorption of the controlled delivery system. Hence, the findings are only applicable to other active ingredients of similar chemistry to MFZ. It is also acknowledged that a synthetic, sandy soil was used to reduce compositional variability; natural field soils possess greater heterogeneity, organic matter content, and microbial activity that can substantially alter the adsorption behavior and the fate of nanoparticles and pesticides. In addition, the influence of environmental factors was assessed using only two pH conditions and two temperature levels, which provide an initial understanding of the system behavior but do not encompass the wide range of environmental conditions encountered in agricultural soils. While this simplified experimental approach enabled systematic evaluation and comparison across two different systems, it does not fully capture the complexity of natural soil systems, where variations in organic matter content, microbial activity, pH, and temperature can substantially influence pesticide adsorption and degradation of LNP systems. Therefore, future studies should be done to evaluate the developed LNP delivery system for a broader spectrum of agrochemicals (fungicides, herbicides, and insecticides) under a broader range of pH and temperature conditions and representative natural soils to understand the practical applicability and conduct a field validation to assess the plant uptake, efficacy against the targeted pests, and impact on nontarget organisms.

These results reported in this work, using MFZ as a model pesticide, demonstrate that LNPs can serve as engineered delivery systems of tunable properties with improved soil retention compared to free pesticides or commercial formulations. Furthermore, the variation in lignin type can be beneficial for engineering delivery systems with controlled properties that modulate MFZ release under different soil pH and temperature conditions and reduce pesticide runoff through enhanced soil adsorption, addressing concerns over agrochemical-induced environmental contamination. Although the transitions from controlled laboratory conditions to agricultural fields require validation across diverse soils, climatic conditions, and targeted pests with efficacy assessments, the findings of this study demonstrate the strong potential of lignin-based NP systems to offer a sustainable pathway for improving pesticide delivery while reducing overall pesticide use.

## ∥

Department of Chemical and Biomolecular Engineering, Johns Hopkins University, Baltimore 482, United States
